# Regulated Mycotoxin Occurrence and Co-Occurrence in Croatian Cereals

**DOI:** 10.3390/toxins14020112

**Published:** 2022-02-02

**Authors:** Marija Kovač, Mateja Bulaić, Ante Nevistić, Tomislav Rot, Jurislav Babić, Mario Panjičko, Tihomir Kovač, Bojan Šarkanj

**Affiliations:** 1 Department of Food Technology, University North, Trg dr. Žarka Dolinara 1, 48000 Koprivnica, Croatia; marija.kovac@unin.hr (M.K.); bsarkanj@unin.hr (B.Š.); 2 Inspecto Ltd., Industrijska Zona Nemetin, Vukovarska Cesta 239b, 31000 Osijek, Croatia; mateja.bulaic@inspecto.hr (M.B.); ante.nevistic@inspecto.hr (A.N.); tomislav.rot@inspecto.hr (T.R.); 3 Faculty of Food Technology, Josip Juraj Strossmayer University of Osijek, Franje Kuhača 18, 31000 Osijek, Croatia; jbabic@ptfos.hr; 4 CROTEH–Sustainable Technologies Development Centre Ltd., Dragutina Golika 63, 10000 Zagreb, Croatia; mario.panjicko@croteh.eu

**Keywords:** EU regulated mycotoxins, cereals, co-occurrence, weather conditions, LC-MS/MS

## Abstract

A total of 209 samples of various cereal crops (maize, wheat, barley, rye and oats) grown in Croatian fields during 2016 and 2017 were collected to analyze and determine the occurrence and co-occurrence of EU regulated mycotoxins in cereals (AFB1, AFB2, AFG1, AFG2, DON, FB1, FB2, ZEA, T-2, HT-2 and OTA). The analysis, performed by a validated confirmatory LC-MS/MS method based on a dilute and shoot principle, highlighted *Fusarium* mycotoxins as the main contaminants, often co-occurring in samples from both years (50.0% in 2016 and 33.7% in 2017). DON was found to be the most frequent mycotoxin, present in 72.5% of the 2016 samples and 32.6% of the 2017 samples, while maize proved to be the most contaminated cereal type of both years with FUM as the most abundant mycotoxins, with an average concentration of 1180 µg/kg. Moderate temperatures with periods of high humidity favored the accumulation of DON in wheat samples instead of other *Fusarium* mycotoxins, while similar conditions favored maize contamination with FUM. A total of 8.3% of all the 2016 harvest samples and 7.9% of the 2017 harvest samples were assessed as non-compliant, containing mycotoxins in concentrations higher than the levels set by the EU legislation for food.

## 1. Introduction

Mycotoxins, secondary fungal metabolites of low-dose toxicity, are unambiguous food and feed contaminants, able to enter the food chain at any point from the field to fork chain. Cereal crop contamination with toxigenic fungi and consequently mycotoxins can occur in fields, before or after harvest, during crop manipulation and storage [1,2], thus endangering the world’s still most important source of food [2]. According to the data of the Croatian Bureau of Statistics (https://www.dzs.hr, accessed on 20 January 2022), cereals represent about 60% of all sown land in Croatia. A total cereal production, including the 2 most represented agricultural crops, wheat and maize, amounts for over 3,000,000 t, giving a yield of up to 6.0 and 9.0 t/ha, respectively. Other cereal crop types, such as barley, oats and rye, are far less cultivated, however they are still significant for agricultural production, contributing to roughly 400,000 t.

The most important mycotoxin producers are species of the *Aspergillus*, *Fusarium*, *Alternaria* and *Penicillium* genera [3,4,5,6], each of them having favorable conditions for growth and toxin production. For example, the *Aspergillus* species generally require low water activity and high temperatures, while the *Fusarium* species mostly prefer higher water activity and moderate temperature range [1,7]. Therefore, mycotoxin presence in cereals is greatly influenced by weather conditions, including air humidity and temperature, but is also influenced by crop cultivation measures, such as chemical treatment, harvest and storage practices, affecting the grain kernels’ quality (e.g., mechanical damage) [2,8,9,10]. 

Mycotoxins are considered to be one of the most important diet risks, causing diseases, mycotoxicoses, in both humans and animals [5,11,12]. Therefore, their levels in certain foodstuff and feedstuff are regulated within the European Union (EU) legislation, via the Commission Regulation (EC) No 1881/2006 [13], Commission Recommendation 2013/165/EU [14], Commission Recommendation 2006/576/EC [15] and Directive 2002/32/EC [16], setting the maximum permitted/indicative levels. For agricultural production in general and cereal production, specifically, aflatoxins (AFT)–B1 (AFB1), B2 (AFB2), G1 (AFG1) and G2 (AFG2), deoxynivalenol (DON), fumonisins (FUM)–B1 (FB1) and -B2 (FB2), zearalenone (ZEA), T-2 and HT-2 toxins and ochratoxin A (OTA) are the most significant regulated mycotoxins. 

Climate change effects, such as a long-term change in temperature, precipitation and atmospheric CO_2_ concentrations, have an enormous impact on the incidence and the types of hazards contaminating food [17]. For example, they are leading to an unexpected (increased or reduced) risk of mycotoxin contamination of crops, affecting the cereals based on a geographical distribution, toxigenic fungi and their secondary metabolites [18]. According to the predictions, under the influence of climate change factors, pests and pathogens, including toxigenic fungi, move towards the poles at a speed of about three kilometers per year, causing latitudinal shifts in species distributions [19]. Thus, a higher incidence of *Claviceps* and *Fusarium* species is expected in Northern Europe, so far characteristic of Central Europe, while a higher *Penicillium* and *Aspergillus* species and AFT incidence, so far characteristic of Southern Europe, is expected in Central Europe [20,21]. Furthermore, climate change factors may affect mycotoxin contamination by altering the mycotoxin prevalence among the same fungal species, or by altering the relative amount of free and modified mycotoxin forms. In addition, they may have an indirect impact related to increased drought stress, insect damage to plants, or changes in crop phenology, such as changes in the flowering time and grain maturation, and all of these factors combined affect the mycotoxin production [5,22,23,24,25]. 

Taking all that into account, mycotoxin concentrations need to be regularly monitored, ensuring the food safety in a changing climate, contributing to public health. To the best of our knowledge, there is a lack of a comprehensive research on the occurrence and co-occurrence of all legally regulated mycotoxins in Croatian cereals. Considering the above-mentioned, together with the (varying) weather impacts on mycotoxin incidence [10,26,27,28,29,30,31,32,33], the aim of our study is to investigate the occurrence and the co-occurrence of all EU regulated mycotoxins in unprocessed Croatian cereals, as well as to establish the correlation between the found mycotoxin concentrations and weather features, i.e., rainfall and air temperature, during the two-year study period, in all Croatian counties. Moreover, the obtained data on the incidence of analyzed regulated mycotoxins in cereals from the 2016 and 2017 harvest were used to create heat maps of mycotoxin incidence, and to review grain contamination levels from each Croatian county.

## 2. Results and Discussion

### 2.1. Mycotoxin Occurrence and Co-Occurrence in Cereals

The samples of various cereals grown in Croatian fields during 2016 and 2017 were contaminated with all of the regulated and analyzed fungal metabolites. *Fusarium* mycotoxins proved to be the most common contaminants, found in all the samples in concentrations above LOD. In both analyzed harvest years, 2016 and 2017, DON was found to be the most frequent mycotoxin, appearing in 72.5% and 32.6% of all cereal samples, respectively. In 2016, the highest concentration of DON was found in maize amounting to 4902 μg/kg, while the average value of all positive cereal samples was 595 μg/kg, similar to the average value of the 2017 samples when the mean value was 510 μg/kg. However, the highest measured concentration for the 2017 samples was 2408 μg/kg, found in wheat. The concentrations of EU regulated mycotoxins in all the cereal samples from 2016 and all the cereal samples from the 2017 harvest obtained in this study using the dilute and shoot LC-MS/MS method are presented in Table 1. The obtained mycotoxin occurrence data on the individual crop types from 2016 and from 2017 are shown in Table 2 and Table 3.

A total of 17 cereal samples (10 harvested in 2016 and 7 harvested in 2017) did not comply with the permitted values of mycotoxins for food presented in the Commission Regulation (EC) no. 1881/2006 [13]. One sample (maize) from 2016 and two samples (wheat and oats) from 2017 did not even comply with the permitted values for animal feed set by the Commission Recommendation 2006/576/EC [15]. An overview of non-compliant samples, with the stated counties of origin, is given in Table 4. The most contaminated cereal type from 2016 and 2017 was maize, with FB1 being the most common mycotoxin (contaminating 85.7% of all the maize samples), followed by HT-2 (73.8%), DON (65.5%), FB2 (64.3%), ZEA (54.8%), T-2 (28.6%), AFB1 (2.4%), and AFB2 and AFG1 (1.2%). Mycotoxin co-occurrence was found in 50.0% of all the 2016 samples (98.4% of all the maize samples), containing 2 or more mycotoxins. The highest co-occurrence was observed for *Fusarium* mycotoxins, DON, FB1, FB2, ZEA, T-2 and HT-2, which were contained in 26.2% of all the the maize samples. FB1 and FB2 in maize samples were the most co-occurring mycotoxins in the 2017 samples, followed by DON and HT-2 in wheat samples, giving a total of 33.7% of all the analyzed samples containing 2 or more mycotoxins. An overview of all the co-occurring mycotoxin combinations found in cereals from the 2016 and 2017 harvests is shown in Table 5.

**Figure A1:**
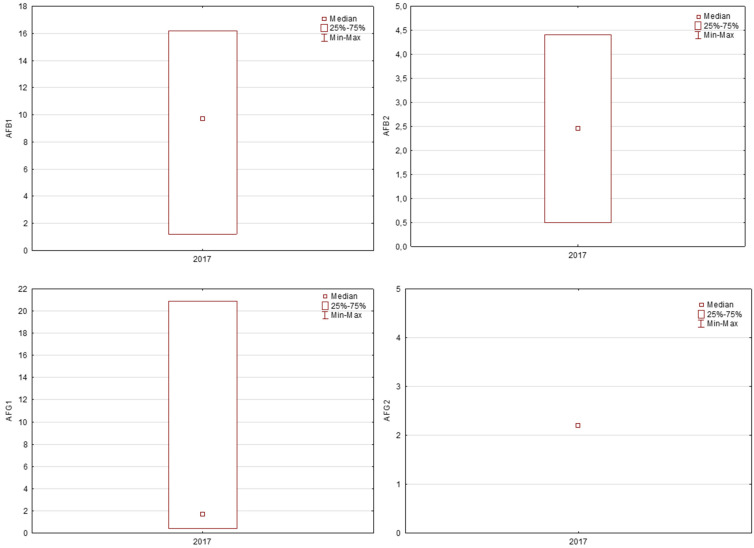
AFB1, AFB2, AFG1 and AFG2 in 2017 harvest cereals.

**Figure A2:**
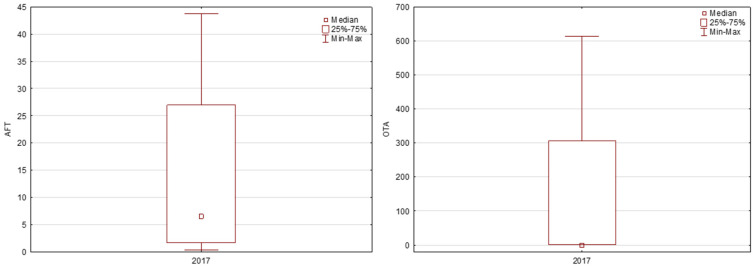
AFT and OTA in 2017 harvest cereals.

**Figure A3:**
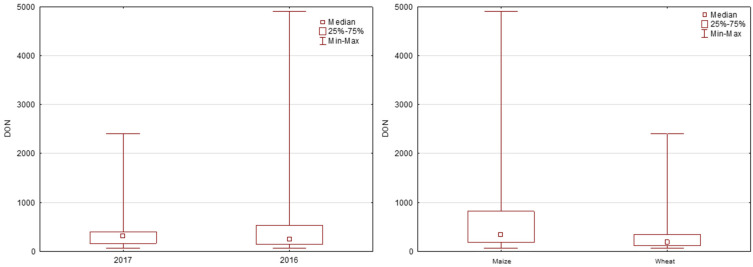
DON in 2016 and 2017 harvest cereals, DON in maize and wheat.

**Figure A4:**
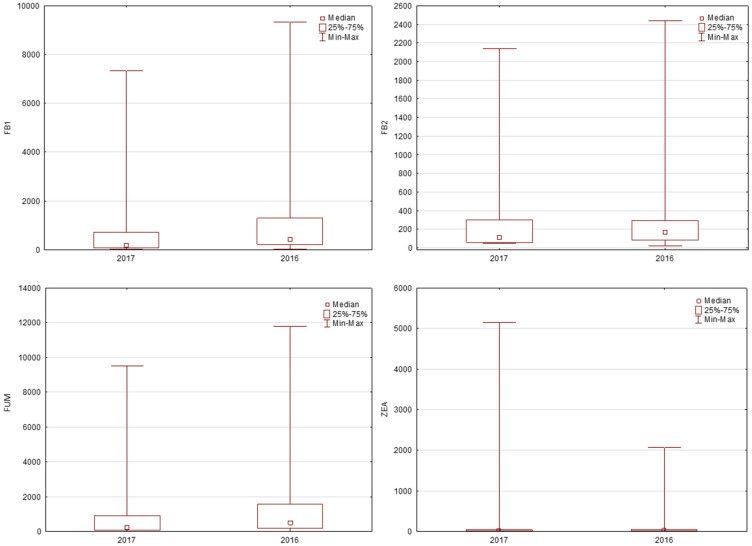
FUM, FB1, FB2 and ZEA in 2016 and 2017 harvest cereals.

**Figure A5:**
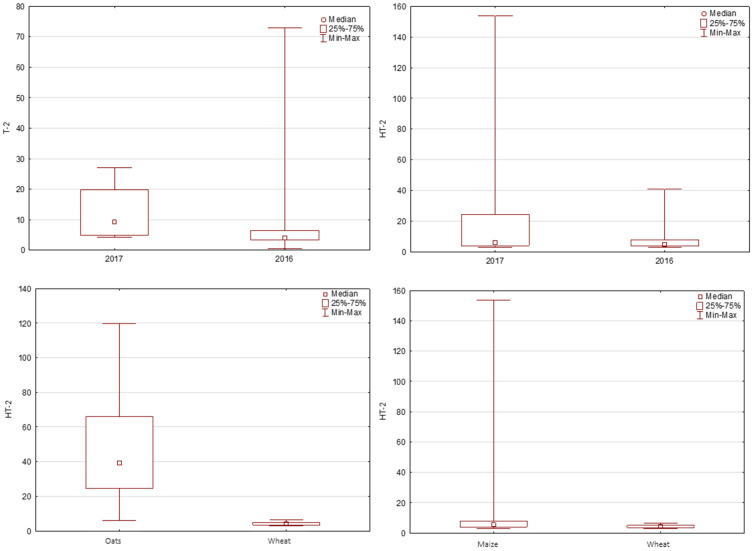
T-2 and HT-2 in 2016 and 2017 harvest cereals; HT-2 in oats and wheat; HT-2 in maize and wheat.

Mycotoxin occurrence data analysis revealed the statistically significant difference in the AFT (*p* = 0), FB1 (*p* = 0.042), FUM (*p* = 0.041)), T-2 (*p* = 0.038) and OTA (*p* = 0) distribution between the harvest years 2016 and 2017, unlike for other tested mycotoxins, for which no statistically significant difference (DON (*p* = 0.740), FB2 (*p* = 0.445), ZEA (*p* = 0.578) and HT- 2 (*p* = 0.139)) was found. A comparison of the variability of mycotoxin contamination between the individual crops showed a statistically significant difference in the occurrence of DON (*p* = 0.002) and HT-2 (*p* = 0.038) between maize and wheat, as well as a statistically significant difference in the HT-2 concentration (*p* = 0.002) between wheat and oats, which was expected, given the focus of legislation [14] on the HT-2 toxin (T-2/HT-2 sum) in oats, distinguishing this type of cereal from others. The obtained statistical analysis data are summarized and presented in box-plot diagrams for both years and the analyzed mycotoxins in Appendix A (Figure A1, Figure A2, Figure A3, Figure A4 and Figure A5). 

Data on mycotoxin occurrence and co-occurrence in Croatian cereals reported by other authors for harvest seasons 2016 and 2017 are limited; however, the data that are available for the relevant years agree with the data of the study conducted in this work. Kovač et al. (2021) [2] conducted a multicontaminant analysis of maize and wheat grown in Croatian fields in 2016, including regulated mycotoxin occurrence determination. *Fusarium* mycotoxins were found to be the most significant secondary metabolites, with DON being the most frequent, present in 73.7% of samples, while AFT and OTA were not detected. The analysis also revealed 8.5% of cereal samples non-compliant to the EU legislation for food regarding mycotoxins. Sunic et al. (2021) [34] investigated *Fusarium* mycotoxins levels, including regulated DON and ZEA, in the unprocessed wheat from the seasons in 2019/2020 from two locations (Vukovar-Syrmia and Osijek-Baranja counties), as a part of the study of the impact of *Fusarium* head blight disease on different wheat varieties. The found data showed a low contamination of naturally infected wheat with DON and ZEA (all below EU limits), but also found higher concentrations in locations with higher precipitation (from Vukovar-Syrmia County). Research of regulated and unregulated *Fusarium* mycotoxins in barley varieties from the period 2016–2018, conducted by Habschied et al. (2019) [28], showed a generally low incidence and found significantly lower concentrations of regulated *Fusarium* mycotoxins (DON, FB1, T-2 and HT-2), compared to the EU legislation, e.g., the found average levels of DON for 2016, 2017 and 2018 were 73 µg/kg, 83 µg/kg and 51 µg/kg, respectively. The incidence of T-2 and HT-2 toxins in unprocessed cereals (oats, maize, barley and wheat) during 2017 and 2018 were investigated by Kiš et al. (2021) [10]. The lowest incidence of T-2/HT-2 toxins was observed in wheat (19.2%), while the highest was observed in oats (70.0%) with the highest mean concentration of 88 ± 631 µg/kg. The vary of T-2/HT-2 toxin concentrations between the production years 2017 and 2018 was not statistically significant and found T-2/HT-2 levels were generally lower than the EU indicative levels. The occurrence of major *Fusarium* mycotoxins, including DON and ZEA, was investigated by Španić et al. (2019) [27] in wheat varieties from seasons 2015/2016 and 2016/2017, to search the correlation between *Fusarium* head blight disease and mycotoxin occurrence. The results showed 100% DON occurrence in the used control grain samples (non-inoculated wheat samples), with an average value of 70 μg/kg, while ZEA was not found in any of the analyzed control samples.

Mycotoxin incidence in Croatian cereals of earlier harvest seasons was better investigated. Pleadin et al. (2017a) [35] studied the occurrence of AFB1, OTA, ZEA, DON and FUM in samples of unprocessed cereals harvested in 2015. Maize was found to be the most contaminated cereal with 93.9% positive samples for DON, 100% for ZEA, 93.9% for FUM, 16.2% for OTA and 10.8% for AFB1. A total of 46.0% of the maize samples, 4.0% of wheat samples, 8.0% of barley samples and 11.0% of rye samples did not comply with the EU legislation requirements. As a part of *Fusarium* head blight disease research, Španić et al. (2018) [26] conducted a study on wheat varieties from seasons 2014/2015. Naturally contaminated wheat contained certain *Fusarium* mycotoxins, primarily DON, occurring in 64.0% of the analyzed wheat samples. The presence of DON, ZEA, FUM and T-2 toxin in cereals (maize, wheat, barley and oats) harvested in 2011 was investigated by Pleadin et al. (2013) [31]. Maize was proved to be the most contaminated cereal type, with DON as the most occurring mycotoxin (in 52.5% of samples), followed by ZEA (40.5%), FUM (37.5%) and T-2 toxin (33.0%). The authors associated the relatively low mycotoxin contamination with the very warm and dry weather during the observation period, which is supported by the studies [29,30] conducted on cereals from the year 2010, which abounded in rain and cold weather, revealing the significant contamination of the analyzed samples with *Fusarium* mycotoxins.

Furthermore, high air temperature and low amounts of precipitation were correlated to the high occurrence of AFB1 in cereal samples harvested in the years 2009–2013, and in products thereof [32,33], emphasizing the importance of the geographical origin of the crops, i.e., the weather conditions.

The obtained data also conform to the mycotoxin occurrence and co-occurrence studies from nearby/Balkan countries, with a climate similar to Croatia. In the Serbian study conducted by Radić et al. (2021) [36], it was shown that the growing season had an impact on *Fusarium* mycotoxin occurrence in maize; generally higher levels of certain *Fusarium* mycotoxins were detected in 2016, compared to the 2017 samples, due to increased humidity. Among the analyzed *Fusarium* metabolites in maize, FUM were found to be the most frequent. Stanciu et al. (2019) [37] performed a study on trichothecene and ZEA presence in Romanian wheat from the 2015 harvest season. The highest overall frequency was registered for DON, and the authors correlated the mycotoxin concentration with the precipitation and temperature values during anthesis and the preharvest period. Cereal samples from Bosnia and Herzegovina were investigated by Pleadin et al. (2017b) [38] studying the occurrence of certain *Fusarium* mycotoxins. Among the analyzed unprocessed cereals (maize, wheat, barley), maize was the most contaminated, containing DON in 85.2%, ZEA in 73.0% and FUM in 66.9% of the samples. Higher mycotoxin levels determined from the 2014 samples, compared to the samples from 2013 and 2015, were linked to extreme humidity observed during the cereal growth and harvesting periods [37].

### 2.2. Weather Conditions and Mycotoxin Concentrations in Cereals

**Figure 1:**
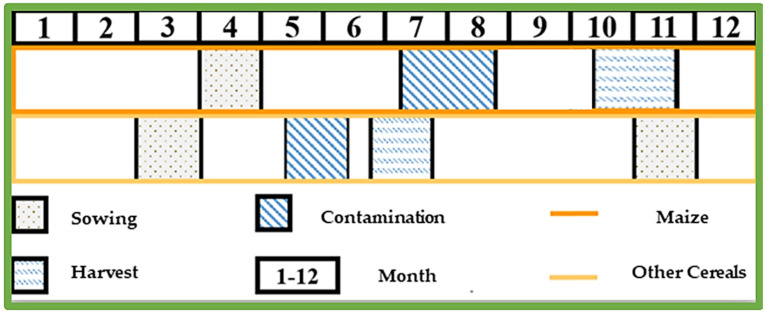
Sowing, contamination and harvest periods for certain cereal crops.

Given the above, mycotoxin biosynthesis is greatly influenced by climatic conditions, humidity and temperature, contributing to the mycotoxigenic fungi colonization and mycotoxin production [1,7,10,39]. The plant is generally most susceptible to contamination during the flowering period; in the case of maize, the plant is most susceptible to contamination in late July and early August (the anthesis and the silking period), when *Aspergillus* fungi and consequently AFT contamination is more likely to occur if extreme drought takes place. In addition, the rainy periods during September and October can cause the contamination of maize with *Fusarium* fungi and mycotoxins. In wheat and other spring crops, the end of May and the beginning of June are critical, since extremely rainy periods favor *Fusarium* fungi contamination [26,27]. Figure 1 shows the sowing, contamination and harvest periods of certain cereal crops.

Relatively low average levels or the absence of found mycotoxins in wheat and other spring crops from both 2016 and 2017 can be explained by the unfavorable weather conditions during crop flowering, generally characterized by relatively mild temperatures and low amounts of precipitation. According to the publicly available meteorological data, temperature conditions in May and June of both years were normal to very warm, while precipitation conditions were normal to wet, both not deviating significantly from the multi-year average. The incidence of DON in wheat samples (68.4% in 2016 and 42.6% in 2017) instead of other *Fusarium* mycotoxins, is likely to be caused by moderate temperatures with periods of high humidity that helped certain *Fusarium* fungi contamination and DON accumulation [40], which is confirmed by the general absence or low contamination of cereals by ZEA. The sporadic high concentration of ZEA found in the oat sample (5155 μg/kg, non-compliant sample) from Osijek-Baranja County was probably caused by somewhat colder (normal) temperature conditions during May 2017, compared to the rest of the country, as well as by improper storage, since other samples from the same county were within the EU permitted levels. Moreover, the average monthly air temperatures, mostly below the multi-year average in October 2016, may be the cause of the significant ZEA production of *Fusarium* species and maize contamination in the 2016 harvest samples. Weather conditions, i.e., temperature and precipitation, during September and October 2016 and 2017 favored the production of FUM and thus the significant contamination of maize (FB1 in 85.7% of all maize samples). High amounts of precipitation in October 2016, which was above the multi-year average, unlike the below-average precipitation in October 2017, may be the cause of higher FUM concentrations found in the 2016 harvest samples (FB1 61–9344 μg/kg and FB2 49–2442 μg/kg), compared to 2017 (FB1 52–7350 μg/kg and FB2 47–2139 μg/kg). The high concentration of AFT found in the 2017 maize sample from the Split-Dalmatia County (AFB1 9.7 μg/kg and AFB2 0.5 μg/kg) can be explained by the high temperatures during the silking period, i.e., a very warm July and extremely warm August throughout the country and drought in the southern regions, which probably favored the development of aflatoxigenic fungi and the production of AFT. For comparison, in the 2016 harvest samples, AFT was not detected in any of the analyzed samples, and according to the available meteorological data, August 2016 is characterized by normal precipitation amounts in most of the country and normal to warm temperatures that did not favor the occurrence of these secondary metabolites. Significant contamination of a wheat sample originating from Šibenik-Knin County with AFT (AFB1 16.2 μg/kg, AFB2 4.4 μg/kg, AFG1 20.9 μg/kg and AFG2 2.2 μg/kg), but also the unusually high OTA presence (614 μg/kg) in the same sample, cannot be explained by weather conditions at the certain micro location, thus it can be assumed that is probably a consequence of accidental contamination due to contaminated storage space. Additionally, the presence of G group AFT (AFG1, AFG2) indicates crop contamination with *A. parasiticus*, which is not a common resident of cereal fields [5,11,41]. 

**Figure 2:**
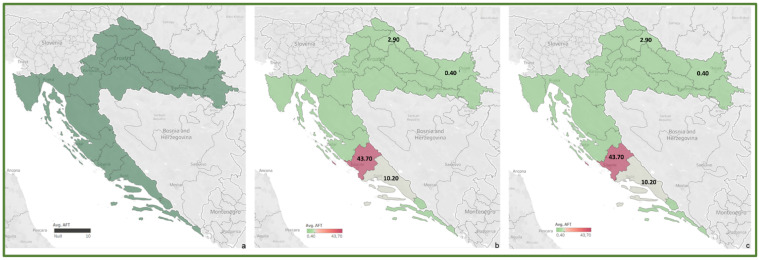
Heat maps of AFT occurrence in Croatian cereals from the harvests of 2016 (**a**), 2017 (**b**) and 2016–2017 (**c**). The range of found concentrations of AFT (legend Avg. AFT) is shown in green (low concentration) and purple (high concentration). In 2016, (**a**) AFT was not found, while in 2017 (**b**) it was found in concentrations 0.40–43.70 µg/kg.

**Figure 3:**
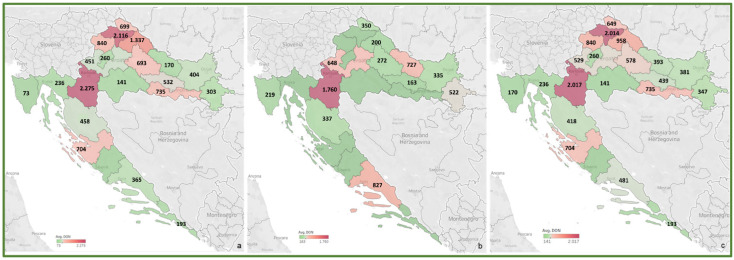
Heat maps of DON occurrence in Croatian cereals from the harvests of 2016 (**a**), 2017 (**b**) and 2016–2017 (**c**). The range of found concentrations of DON (legend Avg. DON) is shown in green (low concentration) and purple (high concentration). In 2016, (**a**) DON was found in concentrations 73–2275 µg/kg, while in 2017 (**b**) in concentrations 163–1760 µg/kg.

**Figure 4:**
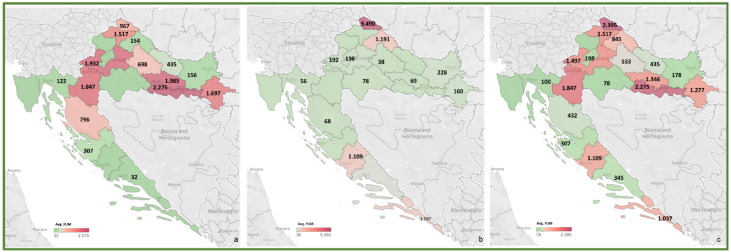
Heat maps of FUM occurrence in Croatian cereals from the harvests of 2016 (**a**), 2017 (**b**) and 2016–2017 (**c**). The range of found concentrations of FUM (legend Avg. FUM) is shown in green (low concentration) and purple (high concentration). In 2016, (**a**) FUM was found in concentrations 32–2275 µg/kg, while in 2017 (**b**) in concentrations 38–9490 µg/kg.

**Figure 5:**
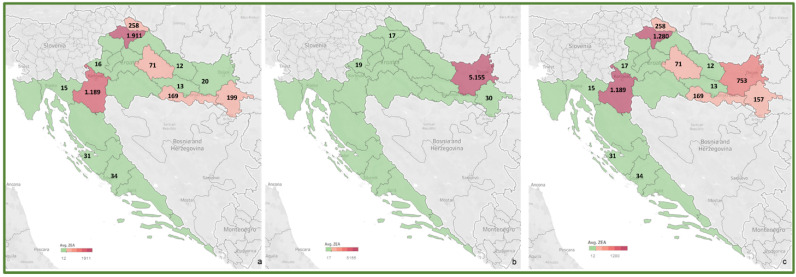
Heat maps of ZEA occurrence in Croatian cereals from the harvests of 2016 (**a**), 2017 (**b**) and 2016–2017 (**c**). The range of found concentrations of ZEA (legend Avg. ZEA) is shown in green (low concentration) and purple (high concentration). In 2016, (**a**) ZEA was found in concentrations 12–1911 µg/kg, while in 2017 (**b**) in concentrations 17–5155 µg/kg.

**Figure 6:**
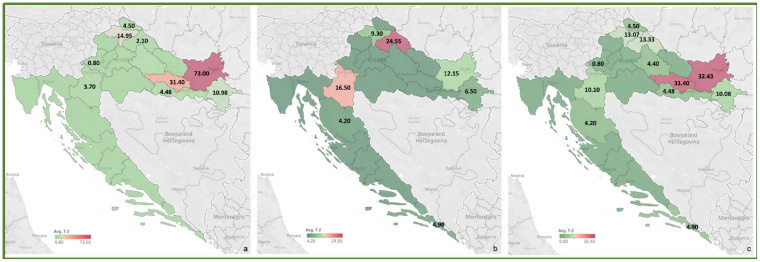
Heat maps of T-2 occurrence in Croatian cereals from the harvests of 2016 (**a**), 2017 (**b**) and 2016–2017 (**c**). The range of found concentrations of T-2 (legend Avg. T-2) is shown in green (low concentration) and purple (high concentration). In 2016, (**a**) T-2 was found in concentrations 0.80–73.0 µg/kg, while in 2017 (**b**) in concentrations 4.20–24.55 µg/kg.

**Figure 7:**
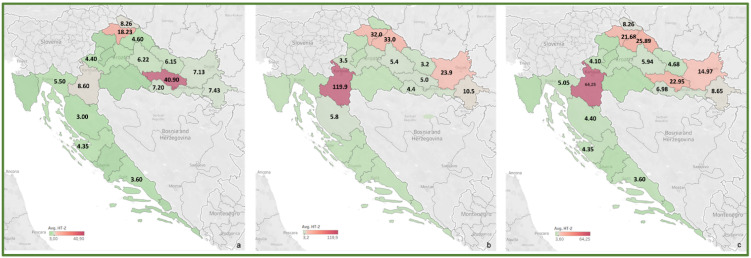
Heat maps of HT-2 occurrence in Croatian cereals from the harvests of 2016 (**a**), 2017 (**b**) and 2016–2017 (**c**). The range of found concentrations of HT-2 (legend Avg. HT-2) is shown in green (low concentration) and purple (high concentration). In 2016, (**a**) HT-2 was found in concentrations 3.00–40.90 µg/kg, while in 2017 (**b**) in concentrations 3.2–119.9 µg/kg.

**Figure 8:**
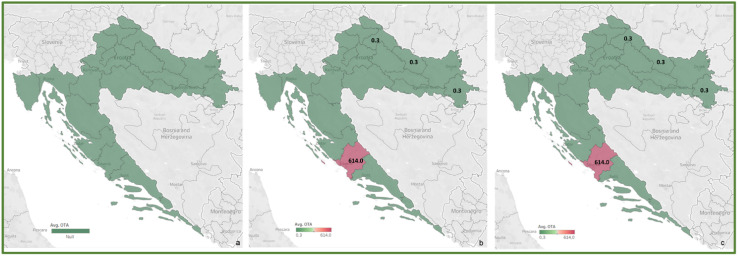
Heat maps of OTA occurrence in Croatian cereals from the harvests of 2016 (**a**), 2017 (**b**) and 2016–2017 (**c**). The range of found concentrations of OTA (legend Avg. OTA) is shown in green (low concentration) and purple (high concentration). In 2016, (**a**) OTA was not found, while in 2017 (**b**) it was found in concentrations 0.3–614 µg/kg.

To review the cereal contamination levels of regulated mycotoxins from each Croatian county, the obtained data were used to create heat maps of mycotoxin occurrence (AFT (AFB1, AFB2, AFG1, AFG2), DON, FUM (FB1, FB2), ZEA, T-2, HT-2, OTA) for the harvest years 2016 and 2017, and for the average of 2016–2017, presented in Figure 2, Figure 3, Figure 4, Figure 5, Figure 6, Figure 7 and Figure 8. The range of found mycotoxin concentrations is shown using green and purple colors, representing the concentrations ranging from very low to very high. In both harvest years, 2016 and 2017, DON significantly contaminated samples from almost every Croatian county, while the highest incidence was observed in Karlovac and Varaždin counties in 2016, and Karlovac County in 2017. FUM were present in a large part of continental Croatia in the 2016 samples, with the highest found concentrations in Brod-Posavina County, while a lower incidence of FUM was recorded in the 2017 samples, with an outlier of 9490 μg/kg found in Međimurje County. Significant concentrations of ZEA in the 2016 harvest samples were found in the north and east of Croatia, and the highest concentrations were recorded in Varaždin and Karlovac counties. The same mycotoxin contaminated the cereals in 2017 significantly less, and the highest incidence was determined in Osijek-Baranja County. The occurrence of T-2 toxin in 2016 was the most significant in the eastern part of Croatia (Osijek-Baranja and Požega-Slavonia counties), similar to HT-2, which was found in the highest concentrations in Požega-Slavonia County. In 2017, T-2 and HT-2 most frequently appeared in the Karlovac and Koprivnica-Križevci counties, followed by Varaždin and Osijek-Baranja counties. AFT were found in only a few samples of the analyzed cereals from the 2017 harvest year, with the highest concentrations originating from Southern Croatia (Šibenik-Knin and Split-Dalmatia counties), which also contained a significant concentration of OTA.

The occurrence and found concentrations of dominant *Fusarium* mycotoxins, DON and FUM, in cereals from 2016 and 2017 are correlated with the annual amount of precipitation at certain locations, which is the cause of significant contamination with these mycotoxins in 2016 cereals, compared to 2017. The annual amount of precipitation for 2016 at a greater number of locations was above the multi-year average, than for 2017. DON and FUM occurrences in cereals originating from Northern and Central Croatia with normal annual precipitation amounts can be explained by rainy periods during the flowering period, when crop infection by the *Fusarium* species occurred, while a significant incidence of AFT and OTA in crops in Southern Croatia (areas around the cities of Šibenik, Knin and Split) corresponds to dry annual precipitation amounts during 2017.

## 3. Conclusions

The research conducted in the current study provides the first comprehensive occurrence and co-occurrence data on all mycotoxins regulated in cereals by the EU legislation (AFB1, AFB2, AFG1, AFG2, DON, FB1, FB2, ZEA, T-2, HT-2 and OTA), presenting a clearer insight on the profiles of mycotoxin contamination of samples from all Croatian counties, together with the impact of weather conditions. The results highlighted *Fusarium* mycotoxins as the main contaminants, with DON being the most frequent in all the sampled cereal crops, while maize proved to be the most contaminated cereal type for both years. A total of 8.3% of all the harvest samples from 2016, and 7.9% of the 2017 harvest samples were non-compliant regarding the permitted levels given in the EU legislation for food. Mycotoxin co-occurrence was confirmed in 50.0% and 33.7% of cereal samples from 2016 and 2017, respectively, with *Fusarium* mycotoxin combination being the most frequent. Furthermore, the found mycotoxins and their concentrations are shown to be related to meteorological conditions during the flowering period of the plants, and the reason for the more significant contamination level of the 2016 cereals rather than 2017. Even though a relatively small percentage of the analyzed cereal samples was assessed as non-compliant to the EU legislation for food, sporadically high concentrations of certain mycotoxins, as well as a large number of co-occurring mycotoxin combinations, point out the necessity of the constant and continuous monitoring of mycotoxin levels and gathering enough data for proper toxicological studies, to ensure food safety in a world exposed to climate change.

## 4. Materials and Methods

### 4.1. Sample Collection

A total of 209 samples of unprocessed cereals (maize 84, wheat 104, barley 9, rye 6 and oats 6) grown in the Croatian fields in the years 2016 and 2017, were selected to analyze and determine the occurrence and co-occurrence of EU regulated mycotoxins. The samples were collected from all Croatian counties, at least one sample from each county and the rest according to the cereal type representation. The sampling was conducted in warehouses and silos in accordance with the Commission Regulation (EC) No 401/2006 [42]. To obtain a homogeneous particle size, samples prepared in test portions (10 kg per sample) were ground using a Romer RAS mill (Romer Labs, Tulln, Austria) with a sieve pore size 0.5 mm, reduced to a laboratory subsample of 500 g using a Retsch Sample Splitter RT 12.5 (Retsch, Haan, Germany) and afterwards stored in a freezer at −18 °C pending analysis. 

### 4.2. Chemicals, Reagents and Standards

All certified mycotoxin standards were supplied by Romer Labs Biopure (Romer Labs, Tulln, Austria), stored according to the manufacturer’s instructions and tempered to room temperature before use. LC-MS grade acetonitrile and methanol were obtained from J.T. Baker (J.T. Baker, Deventer, The Netherlands), while LC-MS grade formic acid and ammonium format were supplied by Sigma–Aldrich (Sigma-Aldrich, St. Louis, MO, USA). Ultrapure water was generated by the Niro VV system (Nirosta d.o.o., Osijek, Croatia).

### 4.3. Mycotoxins Determination

The determination of all the 11 mycotoxins regulated in cereals by the above-mentioned EU legislation (AFB1, AFB2, AFG1, AFG2, DON, FB1, FB2, ZEA, T-2, HT-2 and OTA) was performed by a confirmatory LC-MS/MS method, based on a dilute and shoot principle, previously described by Kovač et al. (2021) [2]. The method was in-house validated according to the relevant EU legislation [42,43] to fulfil the performance criteria and other requirements set for the analytical methods. For identification purposes, the relative retention time of the certain analyte had to correspond to that of the calibration solution at a tolerance of ±2.5%, and the relative ion intensities (quantification and confirmation ion response ratio) had to match an amount of ±30% of the ion ratio in the analyte standard from the same measurement sequence, thus satisfying the criteria of the Commission Decision (EC) No. 657/2002 [43]. The external matrix-matched calibration for each cereal type was used for quantification purposes, while internal quality control was conducted by performing the recovery experiments by spiking within each sample batch, correcting the measured concentrations for recovery when outside the allowed range (90–110%) for mycotoxins by the Commission Regulation (EC) No. 401/2006 [42]. An additional method control was carried out via participation in proficiency testing schemes in both years, in which measurements were taken and evaluated using the obtained z-scores (presented in Appendix B, Table A1), which were considered as satisfactory when within range |z| ≤ ±2.

### 4.4. Statistical Analyses

Statistical software packages, Statistica 13.3 (*TIBCO* Software Inc., Palo Alto, CA, USA) and Tableau 2019.4.5 (3 Excel 2016 (Microsoft, Redmond, WA, USA), were used for statistical data processing. In results processing, the methods of descriptive statistical analysis were used: measures of central tendency and measures of variability (range, standard deviation) calculated from the obtained measurements. The normality of the data distribution was tested by the Shapiro–Wilk W test, the homogeneity of the Levene′s test variance, and a comparison between the two individual variables by the Mann–Whitney U test in data not normally distributed and by the *t*-test in normally distributed data. The Spearman′s correlation coefficient was used to calculate the correlation; data with *p* < 0.05 values were considered statistically significantly different.

### 4.5. Meteorological Data/Heat Maps of Mycotoxin Incidence

A comparison of the type and concentration of found mycotoxins with weather conditions at individual micro locations was performed, using the data obtained from the Croatian Meteorological and Hydrological Service (publicly available at https://meteo.hr, accessed on 22 December 2021) on weather conditions (average monthly air temperature, average monthly amount of precipitation) during the cereal growth and harvest season in the years 2016 and 2017. Heat maps of mycotoxin incidence throughout the country (representing each county) were designed using Tableau 2019.4.5 (Tableau Software, Seattle, WA, USA).

### 4.6. Sample Compliance

The compliance of the analyzed samples regarding the mycotoxins was proved by comparing the found mycotoxin concentrations with the permitted/indicative levels for mycotoxins stated in the EU legislation given by the Commission Regulation (EC) No 1881/2006 [13] and the Commission Recommendation 2013/165/EU [14] for food, and the Commission Recommendation 2006/576/EC [15] and Directive 2002/32/EC [16] for feed.

## Figures and Tables

**Table 1:** Mycotoxin occurrence in Croatian cereals of harvests from 2016 and 2017.

Analyte	2016 (*n* = 120)		2017 (*n* = 89)		LOD µg/kg	LOQ µg/kg
	F%	Mean ± SD µg/kg	F%	Mean ± SD µg/kg		
AFB1	n.d.	-	3.4	9.0 ± 7.5	0.3	1.0
AFB2	n.d.	-	2.3	2.5 ± 2.8	0.15	0.5
AFG1	n.d.	-	2.3	11.3 ± 13.3	0.3	1.0
AFG2	n.d.	-	1.1	2.2	0.15	0.5
DON	72.5	595 ± 971	32.6	510 ± 579	61	200
ZEA	35.8	217 ± 484	5.6	1050 ± 2295	9	30
FB1	42.5	1063 ± 1516	23.6	724 ± 1566	46	150
FB2	32.5	280 ± 395	15.7	292 ± 543	46	150
T-2	16.7	12 ± 17	10.1	13 ± 9	3	10
HT-2	45.0	8 ± 8	31.5	22 ± 36	3	10
OTA	n.d.	-	4.5	154 ± 307	0.3	1.0

*n*—number of samples; F%—frequency, number of positive samples with a mycotoxin concentration above LOD; SD—standard deviation; LOD/LOQ—limit of detection/quantification and n.d.—not detected (below LOD).

**Table 2:** Occurrence of regulated mycotoxins in Croatian cereal samples from the 2016 harvest.

Analyte	Maize (*n* = 61)		Wheat (*n* = 57)		Barley (*n* = 2)	
	F% (*n*)	Mean Conc. µg/kg (Min.–Max. Conc.)	F% (*n*)	Mean Conc. µg/kg (Min.–Max. Conc.)	F% (*n*)	Mean Conc. µg/kg (Min.–Max. Conc.)
AFB1	n.d.	n.d.	n.d.	n.d.	n.d.	n.d.
AFB2	n.d.	n.d.	n.d.	n.d.	n.d.	n.d.
AFG1	n.d.	n.d.	n.d.	n.d.	n.d.	n.d.
AFG2	n.d.	n.d.	n.d.	n.d.	n.d.	n.d.
DON	78.7 (48)	865 (68–4902)	68.4 (39)	263 (63–867)	n.d.	n.d.
FB1	83.6 (51)	1062 (61–9344)	n.d.	n.d.	n.d.	n.d.
FB2	65.6 (40)	58 (49–2442)	n.d.	n.d.	n.d.	n.d.
ZEA	73.8 (43)	221 (10–2068)	n.d.	n.d.	n.d.	n.d.
T-2	32.8 (20)	12 (3–73)	n.d.	n.d.	n.d.	n.d.
HT-2	88.5 (54)	8 (3–41)	n.d.	n.d.	n.d.	n.d.
OTA	n.d.	n.d.	n.d.	n.d.	n.d.	n.d.

*n*—number of samples; F%—frequency, number of positive samples with mycotoxin conc. above LOD—limit of detection; and n.d.—not detected.

**Table 3:** Occurrence of regulated mycotoxins in Croatian cereal samples from the 2017 harvest.

Analyte	Maize (*n* = 23)		Wheat (*n* = 47)		Barley (*n* = 7)		Rye (*n* = 6)		Oats (*n* = 6)	
	F% (*n*)	Mean Conc. µg/kg (Min.–Max.)	F% (*n*)	Mean Conc. µg/kg (Min.–Max.)	F% (*n*)	Mean Conc. µg/kg (Min.–Max.)	F% (*n*)	Mean Conc. µg/kg (Min.–Max.)	F% (*n*)	Mean Conc. µg/kg (Min.–Max.)
AFB1	8.7 (2)	5.5 (1.2–9.7)	2.1 (1)	16.2	n.d.	n.d.	n.d.	n.d.	n.d.	n.d.
AFB2	4.4 (1)	0.5	2.1 (1)	4.4	n.d.	n.d.	n.d.	n.d.	n.d.	n.d.
AFG1	4.4 (1)	1.7	2.1 (1)	20.9	n.d.	n.d.	n.d.	n.d.	n.d.	n.d.
AFG2	n.d	n.d	2.1 (1)	2.2	n.d.	n.d.	n.d.	n.d.	n.d.	n.d.
DON	30.4 (7)	484 (72–1156)	42.6 (20)	474 (68–2408)	n.d.	n.d.	33.3 (2)	957 (309–1605)	<LOD	<LOD
FB1	91.3 (21)	724 (52–7350)	n.d	n.d	n.d.	n.d.	n.d.	n.d.	n.d.	n.d.
FB2	60.9 (14)	292 (47–2139)	n.d	n.d	n.d.	n.d.	n.d.	n.d.	n.d.	n.d.
ZEA	13.0 (3)	26 (17–43)	n.d	n.d	n.d.	n.d.	20.0 (1)	17	16.7 (1)	5155
T-2	17.4 (4)	10 (4–27)	n.d	n.d	n.d.	n.d.	33.3 (2)	8 (7–9)	50.0 (3)	20 (17–22)
HT-2	34.8 (8)	18 (5–154)	23.4 (11)	4 (3–7)	14.3 (1)	3	33.3 (2)	139 (32–245)	100.0 (6)	49 (6–120)
OTA	n.d	n.d	8.5 (4)	153.7 (0.3–614)	n.d	n.d	n.d	n.d	n.d	n.d

*n*—number of samples; F%—frequency, number of positive samples with mycotoxin conc. above LOD—limit of detection; and n.d.—not detected.

**Table 4:** Non-compliant cereal samples from the harvests of 2016 and 2017.

Year of Harvest	Cereal	Mycotoxin	Conc. µg/kg	MPL ^1^ µg/kg	MPL ^2^ µg/kg	County	Year of Harvest	Cereal	Mycotoxin	Conc. µg/kg	MPL ^1^ µg/kg	MPL ^2^ µg/kg	County
2016	Maize	FB1	9344			Brod-Posavina	2017	Maize	FB1	7350			Međimurje
		FB2	2442						FB2	2139			
		FB1 + FB2	11,786	4000	60,000				FB1 + FB2	9490	4000	60,000	
	Maize	ZEA	987	350	2000	Vukovar-Syrmia		Maize	AFB1	9.7	5.0	20.0	Split-Dalmatia
	Maize	DON	3853	1750	8000	Brod-Posavina		Wheat	DON	1760	1250	8000	Karlovac
		ZEA	1018	350	2000			Wheat	DON	2408	1250	8000	Virovitica-Podravina
	Maize	DON	3344	1750	8000	Međimurje		* Wheat	AFB1	1.2	2.0	20.0	Šibenik-Knin
		ZEA	966	350	2000				AFB2	4.4			
	Maize	DON	2275	1750	8000	Karlovac			AFG1	20.9			
		ZEA	1189	350	2000				AFG2	2.2			
	* Maize	DON	4232	1750	8000	Varaždin			AFT total	43.7	4.0		
		ZEA	2068	350	2000				OTA	614	5.0	250	
	Maize	DON	3902	1750	8000	Varaždin		Rye	DON	1605	1250	8000	Varaždin
		ZEA	1755	350	2000			* Oats	ZEA	5155	100	2000	Osijek-Baranja
	Maize	DON	4902	1750	8000	Koprivnica-Križevci							
	Maize	DON	2965	1750	8000	Bjelovar-Bilogora							
	Maize	FB1	4681			Zagreb							
		FB2	741										
		FB1 + FB2	5422	4000	60,000								

^1^ Maximum permitted levels for certain mycotoxins in food according to the Commission Regulation (EC) No 1881/2006; ^2^ maximum permitted levels for certain mycotoxins in feed according to Directive 2002/32/EZ and the Commission Recommendation 2006/576/EC; * non-compliant samples according to the legislation for food ^1^ and for feed ^2^.

**Table 5:** Combinations of co-occurring mycotoxins found in Croatian cereals for the harvests from 2016 and 2017.

2016				2017					
Mycotoxin Co-Occurrence	Maize	Wheat	Barley	Mycotoxin Co-Occurrence	Maize	Wheat	Barley	Rye	Oats
DON + FB1 + FB2 + ZEA + T-2 + HT-2	16			FB1 + FB2	8				
FB1 + FB2 + ZEA + HT-2	9			AFB1 + AFB2 + FB1 + FB2	1				
DON + FB1 + FB2 + ZEA + HT-2	8			AFB1 + AFG1 + DON + FB1 + FB2	1				
DON + FB1 + ZEA + HT-2	7			DON + FB1 + FB2	1				
DON + FB1 + FB2 + HT-2	3			DON + FB1 + FB2 + ZEA	1				
DON + FB1 + FB2 + ZEA	3			DON + FB1 + FB2 + ZEA + HT-2	1				
FB1 + HT-2	3			DON + FB1 + T-2 + HT-2	1				
DON + ZEA + HT-2	2			DON + FB1 + ZEA + HT-2	1				
FB1 + FB2 + HT-2	2			FB1 + FB2 + HT-2	1				
DON + FB1 + FB2	1			FB1 + FB2 + T-2	1				
DON + FB1 + FB2 + T-2 + HT-2	1			FB1 + FB2 + T-2 + HT-2	1				
DON + FB1 + T-2 + HT-2	1			FB1 + T-2 + HT-2	1				
DON + FB1 + ZEA + T-2 + HT-2	1			DON + HT-2		3			
DON + ZEA	1			AFB1 + AFB2 + ZEA		1			
FB1 + FB2	1			DON + HT-2 + OTA		1			
ZEA + T-2 + HT-2	1			DON + OTA		1			
				DON + T-2 + HT-2				1	
				DON + ZEA + T-2 + HT-2				1	
				T-2 + HT-2					2
				ZEA + T-2 + HT-2					1
Number of samples	60			Number of samples	19	6		2	3
% of certain cereal type	98.4			% of certain cereal type	82.6	12.8		33.3	50.0
% of all cereals	50.0			% of all cereals	21.3	6.7		2.2	3.4

## Appendix B. External Method Control Carried Out via Participation in Proficiency Testing Schemes

**Table A1:** Proficiency testing results for mycotoxins in cereals.

Proficiency Testing Scheme (2017/2018)	Matrix	Analyte	Z-Score
Romer Labs CSSMY014–M18161DZ (2018)	Wheat	DON	0.3
		ZEA	1.6
Romer Labs CSSMY017-M19411AF (2018)	Maize	AFB1	−1.7
		AFB2	−1
		AFT (sum)	−1.6
		FB1	−0.4
		FB2	−0.4
		FUM (sum)	−0.7
Bipea 03–0531 (2018)	Barley	AFB1	−1.48
		AFB2	0.00
		AFG1	−0.08
		AFG2	0.35
		T-2	−0.21
		HT-2	0.15
Romer Labs CSSMY012–M17161DZ (2017)	Wheat	DON	0.8
		ZEA	−0.3
Romer Labs CSSMY013–M17411A (2017)	Maize	AFB1	−1.2
		AFB2	0.4
Bipea 04–5131 (2017)	Rye	AFB1	0.00
		AFB2	−0.25
		AFG1	−0.20
		AFG2	−0.45
		T-2	0.52
		HT-2	0.30
